# Cavity Adaptation of Water-Based Restoratives Placed as Liners under a Resin Composite

**DOI:** 10.1155/2017/5957107

**Published:** 2017-03-30

**Authors:** Sheela B. Abraham, Maria D. Gaintantzopoulou, George Eliades

**Affiliations:** ^1^College of Dental Medicine, University of Sharjah, Sharjah, UAE; ^2^School of Dentistry, National and Kapodistrian University of Athens, Athens, Greece

## Abstract

*Purpose*. To investigate the cavity adaptation of mineral trioxide (ProRoot MTA/MT), tricalcium silicate (Biodentine/BD), and glass ionomer (Equia Fil/EF) cements used as liners and the interfacial integrity between those liners and a composite resin placed as the main restorative material.* Materials and Methods*. Standardized class I cavities (*n*: 8 per group) were prepared in upper premolars. Cavities were lined with a 1 mm thick layer of each of the tested materials and restored with Optibond FL adhesive and Herculite Precis composite resin. Cavity adaptation of the restorations was investigated by computerized X-ray microtomography. The regions of interest (ROI) were set at the cavity-liner (CL) interface and the liner-resin (LR) interface. The percentage void volume fraction (%VVF) in the ROI was calculated. The specimens were then sectioned and the interfaces were evaluated by reflection optical microscopy, to measure the % length (%LD) of the interfacial gaps. Selected samples were further evaluated by scanning electron microscopy. Statistical analysis was performed by two-way ANOVA and Student-Newman-Keuls multiple comparison test (*a* = 0.05).* Results*. MT showed significantly higher %VVF and %LD values in CL interfaces than BD and EF (*p* < 0.05). No significant differences were found among the materials for the same values at the LR interfaces.* Conclusions*. When used as a composite liner, ProRoot MTA showed inferior cavity adaptation at dentin/liner interface when compared to Biodentine and Equia Fil.

## 1. Introduction

A variety of dental materials have been introduced as liners or bases to provide pulp tissue protection from physical, mechanical, chemical, and biologic irritants related to the restorative procedure. Liners are usually placed in thin films, whereas bases, considered as dentine substitutes, are placed in thicker layers; they are stronger, but less biocompatible, requiring the additional use of a liner in deep cavities. The traditional lining materials include calcium hydroxide, glass ionomer, resin modified glass ionomer, and pure resinous liners with particles releasing therapeutic agents. From the group of base materials, zinc oxide-eugenol and glass-ionomers were the most popular, with the first excluded from resin composite restorations due to the eugenol-induced inhibition of free radical polymerization [1]. Conventional glass ionomer and resin modified glass ionomer cement are widely used due to their ability to adhere to tooth surfaces, fluoride release, and anticariogenic properties [2]. Their ease of use, fast-setting, low coefficient of thermal expansion, and biocompatibility have made them popular as lining materials [3–5].

The evolution of bioreactive calcium silicate cement (mineral trioxide aggregates, tricalcium silicates, etc.) set a landmark in the development of a unique category of materials combining bioactivity, biocompatibility, and strength [6–9].

The original grey MTA (Dentsply, Tulsa Dental Products, Tulsa, OK, USA), a modification of Portland cement, has been introduced in 1993 [10]. Later, a white MTA version was developed to comply with the esthetic demands, which lacked the tetra-calcium aluminoferrite and had reduced aluminate levels in comparison with the grey formula [11, 12]. MTA products are highly recommended for root-end filling, perforation repair, and pulp capping because of their excellent sealing capacity, biocompatibility, and regenerative properties [9, 13, 14]. However, the very slow setting times made these materials difficult in handling and technique sensitive, especially as bases of main restoratives [11]. Biodentine (Septodont), a faster-setting cement based on tricalcium silicate, was then developed exhibiting the same excellent biological properties like MTA [15]. It can be used as a pulp capping, exerting a positive effect on vital pulp cells stimulating reparative dentine formation. Biodentine demonstrates improved mechanical strength and therefore has been proposed as a dentine substitute in sandwich restorations under composite resin fillings [16, 17].

Adaptation of restorative materials to tooth cavity walls and absence of gaps between restorative and lining materials is crucial for the longevity of the restorations [18–20].

The aim of the present study was to evaluate the cavity adaptation of mineral trioxide, tricalcium silicate, and glass ionomer cement used as bases under composite resin restorations. The null hypothesis tested was that there is no statistical significant difference among the materials selected in cavity adaptation.

## 2. Materials and Methods

Two silicate-based materials (BD, MT) and a high viscosity conventional glass ionomer (EF) were selected as lining materials for this study (Table 1). Caries free premolars (*n* = 24) extracted for orthodontic reasons with intact marginal ridge and similar buccolingual/mesiodistal dimensions were used in the study. The teeth were collected after patient's consent, as approved by the University of Sharjah Institutional Review Board protocol (Ref number 141013). The teeth were cleaned and stored in 0.5% chloramine solution at 4°C for one month, until their use. Prior cavity preparation, the crowns of the teeth were thoroughly cleaned with a cleaning paste and a prophy-brush and rinsed with copious amount of tap water.

Standardized class I cavities (3 mm in length, 1.5 mm in width, and 3 mm in depth) were prepared with tungsten carbide burs (#329, Maillefer, Ballaigues, CH) and finished with fine diamonds (Busch, Engelskirchen, D) placed in an air-rotor handpiece driven by a parallelograph, under constant water cooling. The cavity dimensions were verified by a digital caliper (accuracy ± 0.01 mm). The carbide bur was replaced after every three preparations. Teeth were randomly divided into three experimental groups (*n* = 8) assigned to each of the three lining materials selected (Table 1), which were prepared and placed in cavities according to the manufacturers' instructions. BD and MT were applied in the cavity without any surface pretreatment employing a metal applicator (Dycal instrument, Dentsply, Konstanz, D). For EF group, the cavity floor was conditioned (Cavity Conditioner, GC Corp, Tokyo, JP) for 10 s, water rinsed (5 s), and air dried (5 s), prior to the direct application of the cement from the capsule. All teeth with lining materials received a temporary filling material (Telio CS Inlay/Onlay, Ivoclar Vivadent, Schaan, FL) and stored at 100% RH/37°C for 48 h to allow for adequate material setting. Then, the temporary material was removed from the cavities and the excess of the lining material was removed by a diamond finishing bur mounted in high-speed handpiece under copious water coolant, leaving ~1 mm thick material on the pulpal floor as measured with the digital caliper. The lined cavities were rinsed with tap water, air dried for 5 s, treated with a 3-step etch and rinse adhesive system (Optibond FL, Kerr, Orange, CA, USA) according to the instructions, and restored with a 2 mm single layer of a composite resin (Herculite Precis, Kerr, Shade A2). Photopolymerization of the bonding agent (10 s) and resin composite (30 s) were performed with a LED curing unit (Bluephase G2, Ivoclar Vivadent) emitting 1200 mW/cm^2^ light intensity as measured with a LED curing radiometer (Bluephase meter, Ivoclar Vivadent). The restorations were finished with superfine diamond burs (Busch, Engelskirchen) under continuous water spray and stored in water for 1 week at 37°C. All restorative procedures were performed by two skilled operators. All restorations of each experimental group were randomized between the two operators, so that each operator carried out half of the restorations of each experimental group.

The internal cavity adaptation of the restorative materials was then investigated by computerized X-ray microtomography (micro-XCT), employing a scanner (1072 Skyscan, Aartselaar, B) operated under the following conditions: W source, 100 kV accelerating voltage, 98 *μ*A beam current, 14.16 *μ*m pixel size, 180° rotation at 0.45° step, 1.9 s exposure time per step, and 1 mm Al filter. Horizontal tomographic sections were recorded and reconstructed by using the CTAn software (Skyscan). The regions of interest (ROI) were set at the cavity-liner (CL) and liner-resin composite (LR) interfaces, within a zone of 200 *μ*m extending each site of the interface. The percentage void volume fraction (% VVF: the % of the total empty space at each ROI) was calculated with the same software in 3D scan mode.

Following micro-XCT imaging, each specimen was embedded in epoxy resin and longitudinally sectioned at a mesial-distal direction with a microtome (Isomet, Buehler, Lake Bluff, IL, USA) under continuous cooling. Sections were ground/polished with SiC papers (320–1000 grit size) and a felt with 1 and 0.25 *μ*m grit diamond slurry in a grinding/polishing machine (Ecomet, Buehler) under water cooling. The specimens were immersed for 60 s in a sonicated water-bath, to remove surface attached debris, and the entire section of each specimen was examined under a stereomicroscope (M80, Leica, Wetzlar, D) at 10x magnification. Then, a reflected light optical microscope (DM 4000B, Leica) was used to measure the percentage length of interfacial debonding (%DL) at the cavity-liner (CL) and liner-resin composite (LR) interfaces at 200x magnification.

Representative specimens with and without interfacial defects, as determined by the reflected light optical microscope, were further examined at higher magnification employing a scanning electron microscope (Quanta 200, FEI, Hilsboro, OR, USA), operated in low vacuum mode (LV-SEM) under the following conditions: 20 kV accelerating voltage, 90 *μ*Α beam current, 133 Pa pressure, backscattered electron detector (SSD) in atomic number contrast mode (compositional mode), and 600x magnification.

The results of the %VVF and %DL (independent variables: material and region) were analyzed by 2-way ANOVA on Ranks and Student-Newman-Keuls multiple comparisons test using SigmaPlot 12.3 software (Systat Software Inc., San Jose, CA, USA). An *a* = 0.05 confidence level was selected for all comparisons.

## 3. Results

**Figure 1:**
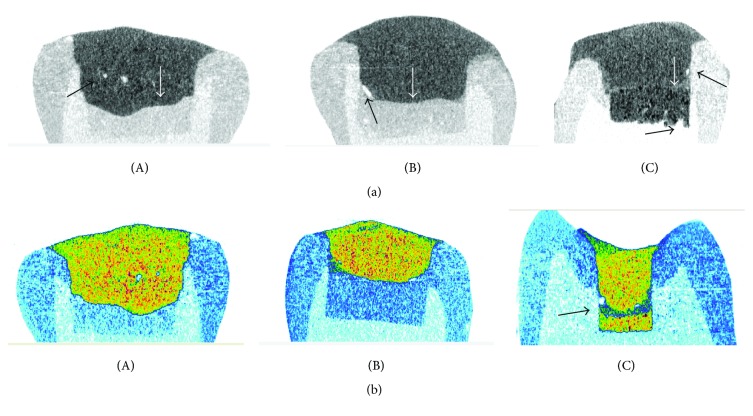
Vertical sections of 2D micro-XCT reconstructed images of BD (A), EF (B), and MT (C). (a) (Grey scale images) white arrows show the composite-cement interfaces and black arrows the presence of interfacial and bulk porosity. More distinct composite-cement interfaces are imaged in BD and EF groups. MT demonstrated porous defects at the cement/dentine interfaces. (b) (Colored images) note the defects at the MTA-composite interface (arrow).

Representative vertical sections from 2D micro-XCT reconstructions of the specimens are presented in Figure 1 (A–C). The interfaces were more clear in specimens lined with BD and EF. In these specimens limited porosity was found at the cement-composite interface or in bulk composite. The interfaces of MT with the pulpal dentine wall and the composite were irregular and noncontinuous with porosity at the cement-pulpal wall interface.

The results of the percentage void volume fraction (%VVF) of the materials tested at the cavity-liner (CL) and liner-resin composite (LR) interfaces are presented in Table 2. The 2-way ANOVA analysis revealed statistically significant difference for both independent factors (*p* < 0.05) and a statistically significant interaction between material and interface (*p* = 0,032). The rankings of the statistically significant differences between the materials were MT > EF, BD for the cavity-liner (CL) interfaces and EF, BD > MT for the liner-resin composite (LR) interfaces (*p* < 0.05). Comparison of the %VVF between the interfacial locations per material showed significantly higher values at the liner-resin composite (LR) interface for BD and EF (*p* < 0.05), but statistically insignificant differences in MT (*p* > 0.05).

**Figure 2:**
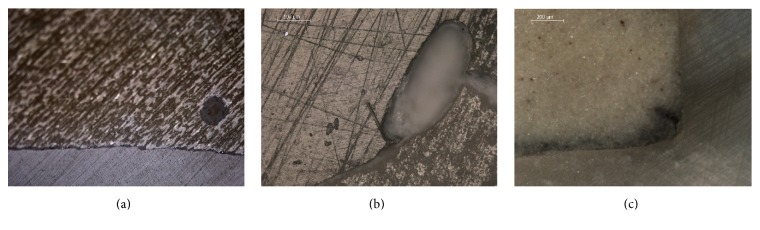
Reflected light microscopic images of cross-sectioned specimens of BD with dentine (a), composite with EF (b), and MT with dentine (c) used for evaluation of the percentage debonding length at the cavity-liner and liner-resin composite interfaces.

Reflected light microscopic images of the cross-sectioned specimens are illustrated in Figures 2(a), 2(b), and 2(c). The results of the percentage debonded length (%DL) at the cavity-liner (CL) and liner-resin composite (LR) interfaces are summarized in Table 2. Again, the 2-way ANOVA analysis revealed statistically significant difference for both independent factors (material and interface, *p* < 0.05) and a statistically significant interaction between them (*p* = 0.004). The ranking of the %DL at the CL interface was similar to %VVF (MT > EF, BD, *p* < 0.05) but showed no statistically significant differences at LR (*p* > 0.05). Comparison between the interfacial locations (CL versus LR) showed statistically significant difference only in BD, with LC exhibiting more than twice the value of LR.

**Figure 3:**
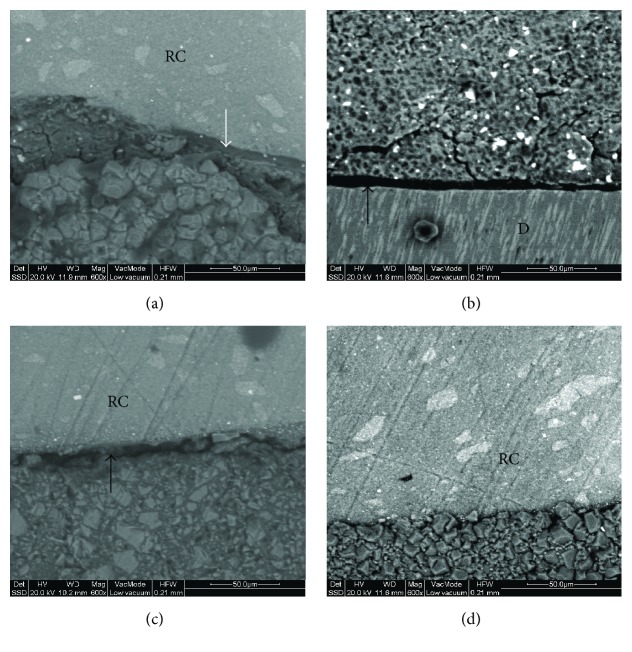
Backscattered images of representative interfaces of the lining materials with dentine (D) and resin composite (RC): (a) BD-composite, (b) BD-dentin, (c) EQ-composite, (d) MT-composite (600x, bar 50 *μ*m). Black arrows show interfacial gaps and white arrow shows the layer of the adhesive.

Backscattered electron images (SSD) of representative specimens at regions of interest identified by the reflected optical microscope are presented in Figures 3(a), 3(b), 3(c), and 3(d). Interfacial defects were mostly related to adhesive debonding at both interfaces.

## 4. Discussion

The results of the present study demonstrated significant differences among the systems tested in the cavity adaptation at dentin-liner and liner-composite interfaces. Therefore, the testing hypothesis was rejected.

Good adaptation of the restorative material to the walls of the cavity and adequate marginal sealing have been considered mandatory for the longevity of a restoration. Marginal gap formation is related to discomfort in conjunction with occlusal forces, which may be attributed to fluid accumulation within the gap and the subsequent fluid movement within the tubules [21], or could also be as a result of shrinkage at the margins as a result of polymerization. The use of 3D analysis of polymerization shrinkage of a dental composite and the resulting gap formation has also been performed using micro-XCT [22, 23]. Microleakage is one of the consequences for restoration failures as it induces sensitivity, leads to colonization of marginal openings by microorganisms, and may lead to recurrent caries and pulpal disease [24].

Several in vitro methods have been applied for interfacial gap assessment. Direct assessment of outer restoration margins is usually performed by reflection optical microscopy [25], confocal microscopy [26], and environmental scanning electron microscopy [27]. Indirect assessment involves evaluation of the interfacial dye penetration or contract agents in microleakage studies. Indirect microleakage evaluation suffers from inherent limitations as the type, size, and concentration of the tracer, the pH of the immersion solution, the chemical affinity of the tracer with the hard dental tissues and the restorative material, and the stain stability [18]. On the contrary, direct imaging techniques are gaining more insight recently.

In the present study cavity wall adaptation assessment was based on the nondestructive three-dimensional (3D) imaging capacity of high resolution micro-XCT. In dental research, micro-XCT has been used for studying tooth and root canal morphology, polymerization shrinkage defects, and microleakage [25, 28]. By the use of the micro-XCT, the cavity adaptation of the restorative material and the internal porosity of the restoration can be imaged and quantified [29, 30]. A recent study by Carrera et al. [31] has shown a technique of how leakages in dental restorations can be quantified using micro-XCT, silver nitrate infiltration, and image segmentation. This could identify defects in the adhesive layer or detect interfacial debonding through polymerization shrinkage.

Glass ionomer cement (GIC) adheres chemically to the tooth structures. The factors considered for creating good adhesion are clean surfaces, surface roughness, proper surface tension and wettability, low viscosity, and adequate flow [32]. Although GIC is aqueous systems and wets tooth structure well, it tends to have relatively high viscosity so it cannot adapt readily to cavity wall microstructures. EQ is a conventional high viscosity restorative GIC with improved mechanical properties, very good adaptation, and very low internal and marginal gap formation [33] due to low shrinkage and stress built-up during setting [34]. In a recent study on class 2 primary molar restorations, EQ showed good cavity wall adaptation comparable to an adhesively bonded bulk-fill resin composite restorative and better than a resin modified GI [28]. In a clinical evaluation of the performance of EQ versus a microfilled hybrid composite on class 2 cavities, both restorative materials revealed similar clinical success over a 4-year period [35]. In both the previous experiments mentioned, the GIC was used as a restorative material [28, 35]. As a dentine substitute, traditional GIC has been clinically used as lining material in the open and closed sandwich techniques [36] with a main issue being the optimum treatment of its surface for a durable adhesion with the resin composite [37].

MTA-type materials are highly biocompatible and have been shown to possess antibacterial and antifungal activity due to their alkaline pH [12]. These materials have limited strength as a dentine substitute and difficult handling [38] but demonstrate enhanced sealing capacity [13, 39] and limited solubility [40]. It has been shown that when MTA is placed on dentin, hydroxyapatite crystals grow around the MTA particles and fill the microscopic gap between the material and dentine [41]. However, the major problem of MTA-type materials is the prolonged setting time. This may cause important clinical problems due to inability of the material to maintain shape and support stresses during this period [13].

Biodentine is a new biocompatible bioactive material which may simulate dentine regeneration by inducing odontoblast differentiation from pulp progenitor cells and has been proposed to be used as a lining material under resin composite restorations [42]. It has superior compressive strength values than reinforced zinc oxide-eugenol cement [43], comparative performance to a resin modified GIC regarding microleakage when used as a dentine substitute [17], and better marginal adaptation to dentine in comparison to MTA cement and GIC [44].

The findings of the present study reveal that MT showed significantly higher mean %VVF and %LD values when compared to BD and EF at the cavity-liner interface. The presence of interfacial porosity should be rather attributed to the handling characteristics of the material. The mixed MT material is viscous and does not easily wet and adapt to the dentine cavity surfaces to which it is applied easily. The difficulties associated with the delivery and packing of the material have long been stated [45]. At the liner-resin composite interfaces more porosity was found in BD and EQ by micro-XCT than the reflected light microscopic measurements. This may be attributed to the low resolving capacity of micro-XCT to discriminate the void volume from the volume occupied by unfilled or low-filled adhesive components by radiopaque filler particles [30]. The topography of the liner-resin composite interface was more irregular in MT micro-XCT images, reflecting the difficulties in handling as reported before. The LV-SEM images demonstrated adhesive type debonding at the regions identified with the defects based on the reflected light microscopic images. Although the LV-SEM used was operated at 133 Pa pressure, in comparison with the 10^−4^ Pa of conventional high-vacuum SEMs, the possibility or dehydration artifacts cannot be excluded for all the lining materials tested, which essentially are water-based cement. For this reason the LV-SEM imaging was performed at already defective regions as identified by the reflected light microscopy at ambient conditions. Moreover, backscattered images were acquired, to provide morphology and phase identification capacity.

The presence of interfacial porosity may anticipate problems in interfacial strength. So far the available information is limited. A study by Kaup et al. [46] to compare the shear bond strength of Biodentine, ProRoot MTA, glass ionomer cement, and composite resin on human dentine showed that Biodentine possesses a shear bond strength to dentine comparable to glass ionomer cement, higher than that of ProRoot MTA but lower than composite resins in combination with a dentine adhesive. Tunç et al. [47] evaluated the adhesive properties of MTA and restorative materials by investigating the shear bond strength of 2 resin composites used with two different bonding systems to tooth colored ProRoot MTA. They recommended that composite resins used with total etch one bottle adhesive systems were an appropriate final restoration in contact with MTA.

## 5. Conclusions

1. MT showed significantly higher mean %VVF and %LD values at the dentin-liner interface when compared to BD and EQ which could be attributed to the poor handling characteristics of the material leading to inadequate adaptation.
2. No significant difference was found among the three tested materials at the resin-liner interface.

## Figures and Tables

**Table 1:** The lining materials used in the study.

Material/code	Composition	Manufacturer
Biodentine/BD	Powder: di-, tri-Ca silicate, CaCO_3_, Fe, and Zr oxides Liquid: H_2_O, CaCl_2_, and modified polycarboxylate	Septodont, St Maur-des-Fossés, France,

Equia Fil/EF	Powder: aluminosilicate glass Liquid: H_2_O, polyacrylic acid, and tartaric acid	GC Corporation, Tokyo, Japan

ProRoot MTA/MT	Powder: Portland cement, bismuth trioxide, and gypsum Liquid: water	Dentsply/Maillefer, Ballaigues, Switzerland

**Table 2:** Results of percentage void volume fraction (%VVF) and percentage of debonded length (%DL) at the cavity-liner (CL) and liner-resin composite (LR) interfaces (means and standard deviations in parentheses). Same superscripts show mean values with no statistically significant differences between the materials at the same interface (lower case letters) and for each material between the two interfaces (upper case letters).

Group	%VVF		%DL	
	CL	LR	CL	LR
BD	0.64 (0.15)^a,A^	1.72 (1.10)^a,B^	12.99 (3.20)^a,A^	28.79 (6.47)^a,B^
EF	0.88 (0.15)^a,A^	1.77 (0.92)^a,B^	18.09 (2.67)^a,A^	24.44 (10.86)^a,A^
MT	1.64 (0.64)^b,A^	1.50 (0.31)^a,A^	31.55 (6.62)^b,A^	30.20 (7.26)^a,A^
